# Implementing practice guidelines for anxiety disorders in secondary mental health care: a case study

**DOI:** 10.1186/1752-4458-6-20

**Published:** 2012-09-20

**Authors:** Maarten K van Dijk, Marc JPM Verbraak, Desiree B Oosterbaan, Anton JLM van Balkom

**Affiliations:** 1 HSK Group, Oude Oeverstraat 120, 6811 JZ, Arnhem, The Netherlands; 2 Behavioral Science Institute, Academic Centre for Social Sciences, Radboud University Nijmegen, Nijmegen, The Netherlands; 3 Department of Psychiatry, UMC St. Radboud, Overwaal Centre for Anxiety Disorders, Nijmegen, The Netherlands; 4 Department of Psychiatry and EMGO Institute, VU University Medical Center and GGZinGeest, Amsterdam, The Netherlands

**Keywords:** Anxiety disorders, Practice guidelines, Implementation strategy, Tools for implementation

## Abstract

**Background:**

Recent years have seen the large-scale development of clinical practice guidelines for mental disorders in several countries. In the Netherlands, more than ten multidisciplinary guidelines for mental health care have been developed since 2003. The first dealt with the treatment of anxiety disorders. An important question was whether it is feasible to implement these guidelines because implementing practice guidelines is often difficult. Although several implementation interventions have proven effective, there seems to be no ready-made strategy that works in all circumstances.

**Case description:**

The Dutch multidisciplinary guidelines for anxiety disorders were implemented in a community mental health care centre, located in the east of the Netherlands. The centre provides secondary outpatient care. The unit within the centre that specializes in the treatment of anxiety disorders has 16 team members with diverse professional backgrounds. Important steps in the process of implementing the guidelines were analysing the care provided before start of the implementation to determine the goals for improvement, and analysing the context and target group for implementation. Based on these analyses, a tailor-made multifaceted implementation strategy was developed that combined the reorganization of the care process, the development of instruction materials, the organization of educational meetings and the use of continuous quality circles to improve adherence to guidelines.

**Discussion and evaluation:**

Significant improvements in adherence rates were made in the aspect of care that was targeted for change. An increase was found in the number of patients being provided with recommended forms of psychotherapeutic treatment, ranging from 43% to 54% (p < 0.01). The delivery of adequate pharmacological treatment was not explicitly targeted for change remained constant.

**Conclusion:**

The case study presented here shows that the implementation of practice guidelines for anxiety disorders in mental health care is feasible. Based on the results of our study, the implementation model used offers a useful approach to guideline implementation. By describing the exact steps that were followed in detail and providing some of the tools that were used in the study, we hope the replication of this implementation methodology is made more practical for others in the future.

## Background

Recent years have seen the large-scale development of clinical practice guidelines for mental disorders in several countries. Based on a systematic evaluation of the existing scientific literature, these guidelines provide recommendations for clinical practice under specific clinical circumstances. By promoting evidence-based practice, these guidelines are expected to improve the quality of care [1]. The Netherlands was among the first countries to develop guidelines for mental health care, with over ten different multidisciplinary guidelines published since 2003 [2]. The first guideline that was published concerned the treatment of anxiety disorders [3]. Anxiety disorders constitute a highly prevalent group of mental disorders which are known to significantly compromise quality of life [4]. Despite the availability of effective psychotherapeutic and pharmacological treatments in Western countries, a large proportion of patients with anxiety disorders still do not receive an evidence-based form of treatment [5-7]. Implementing guidelines for the treatment of anxiety disorders will change that, but to date little is known on how to achieve effective implementation.

The implementation of guidelines is often a difficult process. From other fields of medicine where there is a longer tradition of producing guidelines for clinical practice, we know that the actual use of these guidelines often lags behind their availability [8]. The same holds true within mental health care. A survey on the use of national guidelines developed for mental health care, conducted among a representative sample of 406 Dutch mental health care professionals in 2009, showed that although 91% of these professionals reported being familiar with these guidelines, only 28% said that they actually used them [9].

From the existing studies on implementing guidelines in mental health care, we know that active intervention is necessary to promote adherence to these guidelines [10]. Two meta-reviews on guideline implementation within mental health care show that studies which use complex multi-faceted interventions produce the best results [10,11]. There is a lack of convincing evidence favouring one type of intervention or a specific combination of interventions when implementing guidelines, however. Based on studies into changing medical care within the somatic health care system, Grol and Grimshaw also conclude that no ready-made implementation strategy is superior in all situations [12]. They therefore suggest an implementation model that is to be tailor-made matching the specific setting and the needs of the target group among which changed behaviour is sought. In our study we use such an implementation model described by Grol and Wensing [13] which, because of its global and transparent structure, offers the possibility to tailor-make the implementation strategy.

This implementation model of Grol and Wensing consists of several steps that help to plan, execute and evaluate implementation systematically. The model suggests beginning by determining the goals for improvement by analysing current practices, and then analysing the context in which a change of practice routines is expected to take place. We developed two practical tools that help to provide input for these two diagnostic steps. One is a set of process indicators to measure guideline adherence. The second is a questionnaire that helps to detect factors that can impede or promote guideline adherence. By describing these diagnostic tools in combination with the resulting interpretation of the steps in the implementation model, we hope to provide an example of a sound implementation methodology that can easily be replicated by others. In our case, following this methodology proved very useful. Implementing the multidisciplinary guidelines for anxiety disorders led to a significant increase in guideline adherence.

## Case description

### The treatment setting

The community mental health centre in which the multidisciplinary guidelines for anxiety disorders was implemented is located in the Dutch provincial town of Almelo which has around 78,000 inhabitants. It also serves the residents from the surrounding rural area with around 60,000 inhabitants. The centre is part of a larger mental health institution, also containing (semi-)clinical facilities, being the main provider for mental health care in the region. The community mental health centre provides outpatient care and is structured according to several disorder-specific units. After being referred to the centre by their general physician, patients undergo a standard clinical interview to determine their diagnosis. According to the primary diagnosis, patients are allocated to the appropriate disorder-specific treatment unit.

The unit responsible for treating patients with anxiety disorders consisted of 16 team members when the study began. At that time, the team included: 1 psychiatrist, 1 psychiatrist in training, 1 clinical psychologist, 1 clinical psychologist in training, 2 psychotherapists, 2 health psychologists, 3 health psychologists in training and 1 junior psychologist waiting to start training, 3 psychiatric nurses, and 1 psychiatric nurse in training. So the team members’ experience as health care professionals ranged from beginners to the very experienced. The mean age of the team members was 35 years (range 24–53).

The goals of the organization were to improve transparency and the quality of care provided by the unit for anxiety disorders by implementing national practice guidelines. Implementing multidisciplinary guidelines for anxiety disorders was pursued against the background of a scientific study that as well as investigating the feasibility of implementing the guidelines, also aimed to assess the effectiveness of working according to the guidelines by using a prospective cohort design. At the start of this study, guideline adherence was too low to be able to make any meaningful comparison between the treatment results of patients that received guideline adherent care and those who did not. Successful implementation was thus a necessary step in performing this cohort study.

The treatment coordinator of the anxiety disorder unit was closely involved with this study and a key figure during the process of implementation. During the course of the study, a PhD student supervised by the treatment coordinator had an average of 16 hours per week available for facilitating the process of guideline implementation and the collection of data.

### Dutch multidisciplinary guidelines for anxiety disorders

The multidisciplinary guidelines for anxiety disorders were first published by the workgroup for anxiety disorders in 2003. Based on a systematic evaluation of the scientific literature, the guidelines provide an overview of the state-of-the-art of care for patients with anxiety disorders, including hypochondriasis, ultimately reflecting the consensus of the expert group. As such, the guidelines for clinical practice are described for adult patients with a DSM-IV diagnosis of panic disorder with/without agoraphobia, social phobia, obsessive compulsive disorder (OCD), generalized anxiety disorder (GAD), posttraumatic stress disorder (PTSD), specific phobia and hypochondriasis.

Overall, the main evidence-based treatment steps recommended for the various anxiety disorders can be summarized as follows. According to the Dutch treatment guidelines for anxiety disorders both psychotherapy and pharmacotherapy count as equally valid treatment options. Recommended psychotherapeutic treatment steps consist of cognitive interventions or specific forms of exposure interventions. In cases of posttraumatic stress disorder, eye movement desensitization reprocessing (EMDR) is also considered a valid first-choice treatment option. The first two or three treatment steps in pharmacotherapy consist of prescribing antidepressant medication. The guideline favors selective serotonin reuptake inhibitors (SSRIs) over tricyclic antidepressants (TCAs). In most cases, if an SSRI produces an insufficient result, switching to a second SSRI is recommended before prescribing a TCA. In cases of social anxiety disorder the third pharmacological treatment step is the prescription of a benzodiazepine or monoamine oxidase inhibitor (MAOI). In cases of obsessive-compulsive disorder that are resistant to treatment, the third step consists of augmentation of SSRI therapy with an atypical antipsychotic.

### Measuring guideline adherence with the use of process indicators

One of the activities in the first phase of our project was the development of a set of process indicators as one of the two practical tools that help to provide input for the diagnostic steps in the implementation model. These indicators were developed [14] to gain an understanding of the degree of guideline adherence before the start of implementation and to monitor changes during the process of implementing the anxiety disorder guidelines. These process indicators reflect the percentage of patients receiving recommended care according to a specific guideline recommendation. However, the various anxiety disorder guidelines published in 2003 contained over 134 recommendations for clinical practice. The goal was to arrive at a workable number of indicators, based on the recommendations most relevant to improving the quality of care. An iterative consensus procedure was followed for this. The first step in developing the set of indicators was to select those recommendations that were based on the highest level of scientific evidence. This meant that only recommendations supported by the results of a systematic review, or by the results of at least two independently performed randomized clinical trials with sufficient sample size, were selected. Excluding four recommendations that related to the timing of a specific intervention, the number of selected recommendations was reduced to 38. These 38 recommendations were then reformulated into a preliminary set of indicators.

For instance, for patients suffering from a panic disorder with agoraphobia, the following recommendation: ‘Exposure in vivo is an extremely effective intervention in the treatment of a panic disorder with agoraphobia. In those cases where avoidance plays an important part in the clinical picture of the disorder, there is no reason to apply a different psychological intervention than exposure in vivo a priori’; was reformulated into the process indicator: ‘The percentage of patients with panic disorder with (moderate) severe agoraphobia, indicated for treatment with exposure in vivo, that is offered exposure in vivo’.

In the next step a group of 18 expert clinicians, all members of institutions participating in the Dutch Knowledge Centre for Anxiety and Depression, were asked to judge this preliminary set of process indicators. The definite set of process indicators contained only the preliminary indicators that were judged relevant to clinical practice by 80% of those expert clinicians, and of which at least 60% said that the aspect of care covered by the indicator needed improvement in clinical practice. In this way, a set of 34 process indicators was obtained. These 34 indicators related almost exclusively to pharmacotherapy or cognitive-behavioural treatment of anxiety disorders. It was these forms of treatment that (besides EMDR for patients suffering from PTSD) survived the selection procedures, based on the level of scientific evidence and relevance to improving the quality of care as assessed by the expert group. This selection of indicators coincides with the first two or three steps of the psychotherapeutic and pharmacotherapeutic branches of the treatment algorithms for the various anxiety disorders. Thus the final selection of process indicators helps to measure whether the first two or three recommended treatment steps have been followed in each condition, if indicated.

For this study, the adequacy of psychotherapeutic treatment steps was assessed not only by looking at the percentage of patients receiving the right sort of treatment method. Three additional parameters were brought into the picture by using three supplementary help indicators, to measure the proper execution of each of these treatment steps. If the recommended psychotherapeutic treatment had been offered, these supplementary help indicators reflected: 1) whether a treatment rationale had also been given; 2) whether the accompanying homework assignments had been provided during at least half the sessions, where relevant, and; 3) whether the minimum recommended number of treatment sessions had been provided. The adequacy of the pharmacological treatment was not only assessed by looking at the prescription of the right category of medication. Here, three types of supplementary help indicators were also used. If the recommended type of medicine was prescribed, these reflected: 1) whether one of the specific drugs mentioned in the guideline had also been chosen (e.g. fluoxetine as one of the recommended SSRIs for patients with panic disorder); 2) whether the recommended dosage was prescribed and; 3) whether the drug was maintained long enough to be able to evaluate effectiveness (to view an English translation of the final set of process indicators, and the corresponding supplementary help indicators see Additional file 1).

### The development of a questionnaire to identify factors that could promote or impede guideline adherence

To gain an understanding of factors that could impede or promote the implementation of the guidelines from the professional’s viewpoint, we developed a questionnaire [15] inspired by the theory of planned behaviour (TPB) [16]. We found inspiration in the example of Rebergen and colleagues [17], who developed a TPB-based questionnaire to examine predictors of adherence to guidelines for occupational physicians treating employees with mental health problems. The aim was to develop yet another tool, alongside the set of process indicators, that could be used in the diagnostic phase of implementing the multidisciplinary guidelines for anxiety disorders in other settings too.

To simplify, the TPB states that: 1) if people’s attitude towards the suggested behaviour is more positive; 2) if they think that significant others want them to adopt the behaviour (subjective norm); 3) if they believe they are able to adopt the behaviour this results in; 4) then they will have a stronger intention (motivation) to adopt that behaviour, which makes it more likely that they will actually behave in that way. According to our application of the TPB, the target behaviour is adherence to the Dutch practice guidelines for anxiety disorders by the health care professional. We expected that knowledge of the position of the team members regarding each of the four TPB factors would be helpful when choosing concrete interventions to implement these anxiety disorder guidelines. Several items were formulated to measure each of these TPB constructs. To determine item topics relevant for the use with the anxiety disorder guidelines, 7 health care professionals from the anxiety disorder team were interviewed about factors that from their point of view could impede or promote the actual usage of the guidelines. Topics matching the TPB constructs were then reformulated into 58 items. Most of these took the form of concrete propositions, where the respondent was asked to rate his or her level of agreement on a five-point Likert scale (ranging from “I strongly disagree” to “I strongly agree”). Four of these 58 items asked whether the respondent actually possessed a copy of the multidisciplinary guidelines of anxiety disorders or a summary of it; whether the respondent had read the guidelines; and how the respondent rated his or her knowledge of the content of the guidelines. The answer to these questions were needed to assess how much effort should be put into disseminating the multidisciplinary guidelines for anxiety disorders.

To arrive at a more compact version of the questionnaire and establish the reliability of the different subscales, the original 58-item version of the questionnaire was distributed among 89 health care professionals that worked for one of the institutions participating in the Dutch Knowledge Centre for Anxiety and Depression. In analysing the data it was established that we succeeded in developing a reliable scale to measure the intention of the health care professionals to use the guidelines. After removing two items to further improve the reliability of the scale, we derived an ‘intention’ subscale consisting of five items. Subsequently, for the other three TBP based subscales, the five items that showed the best correlation with this intention scale were selected to form the definite scale of the corresponding construct. As such, three additional subscales were formed as follows: 1) ‘Perceived behavioural control’; a scale that reflects the degree to which the health care professional expects to be able to arrange his or her work so that he or she can adhere to the recommendations in the guidelines. 2) ‘Attitude’; a scale that reflects whether someone holds a positive or negative view of using the guidelines. 3) ‘Social pressure’; a subscale that reflects the perceived social normative pressure to adhere to the guidelines. By doing this, together with the four questions about the possession, and knowledge of the guidelines and one question about how often the professional thought that he or she already used the guidelines, we derived a final 25-item version of the questionnaire to assess factors that could influence use of the guidelines by the professional. This 25-item version was considered to be short enough to be used easily in different treatment settings (to view an English translation of this version of the TPB questionnaire, see Additional file 2). Ultimately, it was this 25-item version of the questionnaire that was used within the anxiety disorder team of the community mental health centre of Almelo as part of the second diagnostic step of the implementation model see reference [15] for more details on the development of the questionnaire.

### The process of implementing the guidelines

Following the steps suggested by Grol and Wensing, an implementation programme was designed specifically tailored to the situation in Almelo [13,18]. The subheadings described below summarize the subsequent steps taken. For each subheading a description is given on how the corresponding step was developed for the anxiety disorder team.

#### Step 1: Analysing current practices and determining goals for improvement

We analysed the provision of care within the anxiety disorder team before starting to implement the guidelines by reviewing the medical records of 150 patients suffering from an anxiety disorder or hypochondriasis, who were treated between 2002 and the beginning of 2004. Checklists were used to assess the adherence to the relevant guidelines. The data collected in this way was used to score the set of process indicators described previously.

After reviewing the medical records of these 150 patients and scoring the appropriate indicators, the analysis showed that improvement was most needed on the adequate provision of cognitive interventions and exposure treatment. Relatively large numbers of patients received a positive indication for being offered this kind of treatment. However, only 15 per cent of patients in the case of cognitive interventions and only 17 per cent of patients in the case of exposure interventions did actually receive these kinds of treatment as they should have. A large number of patients that were being offered these kinds of interventions did not receive the corresponding homework assignments and did not undergo enough treatment sessions. So these aspects of care needed improvement. Also, improvement was necessary in the provision of EMDR for patients with PTSD. Compared to the provision of adequate psychotherapeutic treatment as first line of treatment, there was more adequate provision of the first step of pharmacological treatment. The most significant improvement in pharmacological treatment was deemed necessary in the more advanced steps of the medication algorithms, which however concerned only a small number of patients. Improving the number of patients receiving a recommended form of psychotherapeutic treatment was therefore considered to be the primary aim of implementing the guidelines.

#### Step 2: Analysing the context and target group for implementation

After analysing the actual provision of care and setting goals for improvement, we identified factors that could impede or promote guideline adherence at the level of the organization, the level of the health care providers and the level of the patients. A selection of seven team members with different professional backgrounds, were interviewed in depth about their opinion towards the anxiety disorder guidelines and factors that they thought may possibly impede their adoption. In addition, as part of the second step all of the team members were also asked to fill in the final 25-item version of this TPB questionnaire. The purpose of this was to assess the intentions of health care providers in using the guidelines and to get a rough idea of the position of the team on each of the other three TPB constructs.

During the interviews, concerns were raised about two points. First the quality of the unit responsible for diagnosing newly referred patients and devising their treatment plans. The members of the anxiety disorder team had to adhere to this treatment plan to which patients had consented. Therefore it was deemed crucial that these treatment plans had to reach a higher standard and were to be modelled more according to the guideline recommendations. Second the interviewees also had the impression that they treated many patients with complex problems. They thought that the guideline recommendations would be difficult to apply to these patients and that this would impede guideline adherence.

Based on the data gathered using the TPB questionnaire, we were able to conclude that the team members held rather positive attitudes toward the guidelines in general. On the other hand they did not appear to feel much social pressure to follow guideline recommendations. Some of the team members also reported being unfamiliar with the exact content of the guidelines and many of the team members did not feel overly confident about being able to apply the guideline recommendations in daily clinical practice.

#### Step 3: The selection and development of implementation strategies

In step 2 we identified factors and circumstances that could impede or promote guideline adherence. These factors were found at different levels: organizational (the process of care and foremost the intake, diagnosing and choice of the treatment), patients (the matter of informed consent) and professionals (attitude towards the guidelines, the (felt) autonomy and the limited social pressure). Working on the different facets is one of the main characteristics of the overall approach and so we had to direct interventions on each of these facets.

At the organizational level it was obvious to us that some changes had to be made in how the intake procedure was organized. To improve the reliability of the diagnostic process, the idea was to make it compulsory to use the MINI, a semi-structured interview to derive at a DSM-IV diagnosis [19,20], during the intake phase. It was also thought that it would be better if the treatment coordinator of the anxiety disorder team was made responsible for devising treatment plans for patients with an anxiety disorder or hypochondriasis. Consequently, the decision was taken that as soon as the member of the intake team had established an anxiety disorder or hypochondriasis as the primary diagnosis, the patient would be referred to the anxiety disorder unit and scheduled for a meeting with the treatment coordinator to discuss the various treatment options. Doing this was expected to have two important advantages: 1) It would provide the best guarantee that the treatment plan would match the recommendations of the corresponding practice guideline, because the treatment coordinator would be involved in the process of implementing the guidelines; 2) The treatment coordinator would have an impression of all the patients treated within the unit, which could have added value when evaluating the course of treatment for individual patients during the bilateral treatment evaluations which were scheduled regularly. The expectation was that the treatment coordinator would be better able to help identify possible solutions for the problems mentioned by the therapist with applying the guideline recommendations, if he himself had also met the patient. This should lead to better guideline adherence.

At the patients level the intention was also to develop special patient instruction materials to educate the patient about their disorder and the various treatment options recommended in the corresponding practice guideline. This information was to be sent to patients prior to their meeting with the treatment coordinator. It was expected that it would be more difficult for the team members to ignore or overlook the course of action set out in the treatment plan if they knew that the method of treatment recorded in the plan reflected a considered patient choice.

At the level of professionals several interventions were planned to inform, instruct and commit. Two team meetings were organized to discuss the content of the guidelines. Educational materials for health care professionals, such as desktop versions of the guidelines summarizing the most important points of every guideline and the different treatment algorithms were developed. The purpose of this would be to improve the knowledge of the guideline recommendations considered most important for improving the quality of care on the part of the team members involved. We also intended to develop a treatment folder consisting of the psychological evidence-based treatment manuals that would be used the most frequently by the psychologists in the team, so that they would become accessible to all. Here the expectation was that this would make it easier for the psychologist to apply the recommended treatment methods. Several psychologists would also be sent for training in the use of EMDR because too few of the psychologists on the team were skilled in the use of this intervention. Finally, from the start of implementation, the health care professionals would also be asked to use a checklist with guideline recommendations as the basis for the evaluation of the treatment progress of individual patients in the care of the team. This was to help keep the recommended treatment steps clearly in mind when deciding on a subsequent course of action.

#### Step 4: Executing the implementation plan

The health care professionals in the intake team were trained in using the MINI [19,20]. The process of care was reorganized as suggested in step 3. Two team meetings were held to familiarize the health care providers with the content of the guideline. The first meeting was opened by the manager of the community mental health care centre to stress the importance of the implementation project. The development of the guidelines and its recommendations were both discussed at the meeting. It was emphasized that the guidelines had received the approval of the various professional bodies and the patient organization for people with anxiety disorders. Feedback was also given regarding the current provision of care within the anxiety disorder team by presenting the data derived from the medical records, and the goal for the future was explained: increasing the number of patients who receive a recommended form of psychotherapeutic treatment. It was explained that retaining positive scores on the defined supplementary help indicators was important to obtain a positive score on the main process indicator, by providing the corresponding homework assignments and sufficient number of treatment sessions in case of psychotherapeutic treatment. For pharmacological treatment, the importance of a positive score on the parameters covered by the pharmacotherapeutic help indicators was also emphasized.

Before the second meeting, the health care providers were asked to bring examples of patients from their caseload for whom they thought the guidelines would be difficult to apply. The purpose was to reach a consensus about the practical scope of the guideline recommendations and to reach a consensus about what would constitute legitimate reasons for deviating from the recommendations in the guidelines. By discussing the applicability of the guideline recommendations in these ‘complex’ cases, the opinion that the guideline recommendations could not be applied to most of the patients seen by the anxiety disorder team members also became less credible. The aforementioned instruction materials for patients and the different educational materials for the health care professionals in the anxiety disorder team were developed, tested and distributed according to plan. After the first team meeting, an evaluation of the treatment progress of individual patients in the regular team meetings on patient progress was carried out with guideline recommendations clearly in mind. From that moment onwards, during each treatment evaluation the health care provider explained the course of treatment for the patient along the lines of the algorithms and the treatment coordinator would check guideline adherence. This was an aspect of quality assurance for the provision of optimal care. Also, several psychologists were invited to participate in a course about the use of EMDR with patients suffering from PTSD, as specified in the plan.

#### Step 5: Evaluating progress, and adjusting the original implementation plan

The last step of Grol and Wensing’s model [13] consists of continuously monitoring the progress made. Six months after the start of the implementation, a third meeting was held to share experiences using the guidelines, get an impression of the team members’ opinions about the course of the implementation project, and give feedback on the progress made in implementing the guidelines. At the time of this third meeting, most team members still had a positive attitude towards working according to the guidelines. However, some openly complained about having less autonomy and being less satisfied with their job since the start of the project. The impression was given that this was merely due to the increased supervision of their performance. Literature on this subject also shows a significant association between job autonomy and job satisfaction among health care professionals [21]. These signals were taken seriously. The treatment coordinator changed his style of asking about guideline adherence during treatment evaluations within the team, and the good intentions of the team members wanting to follow the guideline recommendations were taken more seriously. They were asked to talk about treatment progress and indicate themselves whether there were any difficulties in applying the guideline recommendations, without the treatment coordinator asking about guideline adherence proactively and most team members were satisfied with this arrangement. The first signals of an increase in the number of patients receiving the recommended psychotherapeutic care became apparent.

After one year, a sample of medical files was taken from fifty patients who had begun treatment after the implementation of the guidelines to evaluate the progress of implementation in greater detail. The data collected showed that the application of specific cognitive-behavioural techniques still seemed to pose a problem for some team members, although an overall increase in guideline adherence could already be discerned. Two additional team meetings were held. One focused on the use of behavioural experiments in cognitive therapy, in which automatic thoughts had already been tested several times with the use of thought records and Socratic dialogue. This resulted in a first shift in the credibility of the anxious thoughts. The other meeting focused on new insight into the mechanisms of exposure treatment. In this meeting, the way this new insight could be translated into concrete homework assignments for patients with the various anxiety disorders was discussed from the start of therapy. The importance of motivating the patient to complete such assignments was emphasized.

Two years after the official start of implementing the guidelines, another review of medical files was carried out for 181 patients referred to the anxiety disorder team after October 2005 for a final evaluation of the implementation efforts. To assess changes in guideline adherence, a cross-section of the medical files from this second group of patients was taken midway through 2008. The original process indicators were scored once again. To increase power, aggregated information from the disorder-specific indicators was used where possible to reflect general changes in adherence to the recommended treatment steps. Table 1 reflects the patient characteristics of those patients included in the reviews of medical files before and after start of the implementation of the guidelines. As Table 2 shows, there is a significant difference in the number of asylum seekers treated within the anxiety disorder unit before and after implementation of the guidelines, probably due to the closure of a nearby refugee centre during that period. Also, fewer patients with OCD were seen for treatment after the start of implementation. Neither of these two variables were shown to be significantly associated with differences in guideline adherence however.

**Table 1:** Socio-demographic and clinical characteristics of patients in the pre- and post-implementation group

	***Pre-implementation group (N = 150)***		***Post-implementation group (N = 181)***		***p***
Age: mean (SD)	34.0	(11.0)	33.9	(11.0)	0.94
Gender (female): n (%)	93	(62.0)	111	(61.3)	0.90
Living alone: n (%)	32	(21.5)	25	(15.7)	0.19
Educational level; elementary school, at max: n (%)	29	(19.3)	22	(13.9)	0.20
Foreign origin: n (%)	44	(29.3)	40	(22.1)	0.13
Asylum seeker: n (%)	24	(16.0)	12	(6.6)	< 0.01
Panic disorder: n (%)	58	(38.7)	71	(39.2)	0.92
Social anxiety disorder: n (%)	25	(16.7)	29	(16.0)	0.87
Obsessive-Compulsive disorder: n (%)	23	(15.3)	14	(7.7)	0.03
Generalized Anxiety disorder: n (%)	11	(7.3)	17	(9.4)	0.50
PTSD: n (%)	30	(20.0)	39	(21.5)	0.73
Specific phobia: n (%)	3	(2.0)	6	(3.3)	0.52
Hypochondriasis: n (%)	0	(0.0)	5	(2.8)	0.07

Table 2 shows the percentage of patients receiving treatment according to the recommended general treatment steps, before and after implementation of the guidelines and the change in percentage over time.

**Table 2:** Guideline adherence in the pre- and post-implementation group

***Guideline recommendation***	***Pre-implementation group (n = 150)***		***Post-implementation group (n = 181)***		***Difference (%)***	***p***
Number of patients indicated for cognitive interventions and the percentage that actually received it: n (%)	124	(15.3)	109	(69.7)	+54.4	<0.01
Number of patients indicated for exposure interventions and the percentage that actually received it: n (%)	81	(17.3)	50	(60.0)	+42.7	<0.01
Number of patients indicated for treatment with EMDR and the percentage that actually received it: n (%)	23	(43.5)	30	(96.7)	+43.2	<0.01
Number of patients indicated for medication step 1 and the percentage that actually received it: n (%)	54	(55.6)	59	(61.0)	+5.4	0.56
Number of patients indicated for medication step 2 and the percentage that actually received it: n (%)	15	(20.0)	20	(45.0)	+25.0	0.12
Number of patients indicated for medication step 3 and the percentage that actually received it: n (%)	11	(45.5)	12	(16.7)	−28.8	0.19

As can be seen in column 4 of Table 2, there were significant changes in the percentage of patients receiving cognitive interventions (+54.4%, p < 0.01) and the percentage of patients receiving the recommended form of exposure interventions (+42.7%, p < 0.01). Additionally, the percentage of patients with posttraumatic stress disorder who were treated with EMDR, if indicated, was significantly higher (+43.2%, p < 0.01). Even though there were changes in the percentage of the patients being given adequate pharmacological treatment, the number of cases indicated for the different consecutive steps was too small to justify further statistical analyses.

## Discussion and evaluation

After drawing up a tailor-made implementation plan and using multifaceted implementation strategies, significant improvements in adherence rates to the Dutch multidisciplinary guidelines for anxiety disorders were found to have occurred. An increase was found in the number of patients being provided the recommended forms of psychotherapeutic treatment, which was the primary aim of the implementation activities. The delivery of adequate pharmacological treatment was not explicitly targeted for change and remained fairly stable. Generally, it seems that we may safely conclude that the implementation of evidence-based practice guidelines for anxiety disorders within mental health care is feasible.

Based on the experiences in our study, the implementation model of Grol and Wensing offers a useful approach to guideline implementation., It helps to plan and execute implementation activities systematically, and helps to develop implementation interventions that match the requirements of the target group. In our study, the factors that improved guideline adherence were interventions aimed at reorganizing the process of care, greater dissemination of knowledge about the guidelines (including the distribution of training materials to aid in following the guideline recommendations), and measures aimed at increasing normative social pressure in favour of adherence to the anxiety disorder guidelines.

The method of implementation described in this study appears to be effective, and can be easily copied by others. The preparation of implementation aids such as desk-top guides and the patient information materials is an idea that could easily be ‘borrowed’ from this study for use elsewhere. The same is true of the format of the team meetings and the training materials used. The questionnaire used to assess the health care providers’ intention of beginning to use the guidelines can also be lend. Instead of the large sample needed for scientific research purposes, in daily practise small samples of about ten medical records can be used as input for the plan-do-check-act cycle to monitor progress in implementing the guidelines.

One limitation of the study is the use of before-and-after design to evaluate the effect of our implementation activities. Without the use of a control group and proper randomization procedures, we cannot conclude definitively that the changes in behaviour that we observed resulted from the efforts aimed at implementing the guideline. They may well simply reflect the passing of time and the fact that the use of guidelines became slowly more established in the Netherlands. Studies by Bauer [10] and Weinmann et al. [11] show, however, that without active efforts to ensure the implementation of a guideline, they will only be marginally adhered to. This casts doubt on the idea that the changes achieved merely reflect a process that would have happened anyway. Nevertheless, a controlled design is necessary to draw more firm conclusions about the effectiveness of the implementation activities such as those described here. Besides the evidence of the feasibility of implementing evidence-based guidelines for anxiety disorders, our results only allow the conclusion that the tailor-made approach presented here seems promising from the point of view of implementing evidence-based practice guidelines within mental health care.

A significant challenge however will be the maintenance of performance rates in the longer term. Health care professionals leaving the team and new personnel starting, and constantly changing organizational priorities will make this a difficult task. The treatment coordinator – a key figure in ensuring proper guideline adherence – left at the end of the study. The impression was that adherence rates dropped slightly after this. It is very important to continue monitoring guideline adherence and provide continuous feedback. With new personnel coming in, it is necessary to hold regular training days such as those held at the start of the implementation. Our impression is that doing so would prove very worthwhile. The multifaceted approach also seems to be specifically relevant in meeting this challenge because of the different factors which intervene and interact: organizational (for instance organizational rules and leadership), professionals (information, education, instruction and commitment) and the patients level (information, participation). Measures have to be taken at the different facets to maintain the necessary change.

## Conclusions

The case study presented here shows that the implementation of practice guidelines for anxiety disorders in mental health care is feasible. After drawing up a tailor-made plan for implementation and using multi-faceted implementation strategies, significant differences were found on those aspects of care that were targeted for change in the community mental health care centre in which this type of guidelines were implemented. The study also shows, however, that it is important to think about ways to maintain changes made in the provision of care in the longer term. An important question remains whether following such anxiety disorder guidelines does indeed lead to better treatment outcomes, as expected. A future publication will report on the relationship between adherence to such guidelines and treatment outcomes, based on the treatment results gained in patients treated in the community mental health care centre after the start of the activities aimed at implementing the guidelines.

## Abbreviations

TPB: Theory of planned behaviour; OCD: Obsessive compulsive disorder; GAD: Generalized anxiety disorder; PTSD: Posttraumatic stress disorder; SSRI: Selective-Serotonin-Reuptake-Inhibitor; TCA: Tri-Cyclic-Antidepressant; SNRI: Serotonin-Norephinephrine-Reuptake-Inhibitor; MAOI: Mono-Amine-Oxidase-Inhibitor.

## Competing interests

The authors declare that they have no competing interests.

## Authors’ contributions

MKD, MJPMV , DBO, and AJLMB conceived and designed the study. DBO, MJPMV and MKD played an active role in implementing the guideline. MKD was responsible for the data collection for the study. MKD also performed all statistical analyses. All authors participated in interpretation of the results. MKD drafted the manuscript. All other authors provided a critical revision of the draft for important intellectual content and all authors read and approved the final manuscript.

## Supplementary Material

Additional file 1 **Table A.** the definitive selection of process indicators for each disorder. Table B. treatment indicators applicable when pharmacotherapeutic treatment is offered. Table C. treatment indicators applicable when a form of exposure is offered. Table D. treatment indicators which are applicable when cognitive therapy is offered. Click here for file

Additional file 2 Questionnaire relating to factors that impede or promote the application of the anxiety disorder guidelines. Click here for file
