# Supplementary material for: Implementing practice guidelines for anxiety disorders in secondary mental health care: a case study
Source: Int J Ment Health Syst. 2012 Sep 20;6:20. doi: 10.1186/1752-4458-6-20 (PMC3499400; doi:10.1186/1752-4458-6-20)
Supplement: Additional file 1 — Table A. the definitive selection of process indicators for each disorder. Table B. treatment indicators applicable when pharmacotherapeutic treatment is offered. Table C. treatment indicators applicable when a form of exposure is offered. Table D. treatment indicators which are applicable when cognitive therapy is offered. [file 1752-4458-6-20-S1.doc]

Additional file 1

**Table A. the definitive selection of process indicators for each disorder**

| *Panic disorder* | *Percentage of patients with a panic disorder with co morbid severe depression and an indication for treatment with an antidepressant, who have been prescribed one of the antidepressants recommended in the guideline first of all* |
| --- | --- |
|  | *Percentage of patients with a panic disorder with (moderately) severe agoraphobia with an indication for treatment with an antidepressant and who were prescribed one of the antidepressants recommended in the guideline first of all* |
|  | *Percentage of patients with a panic disorder with an indication for treatment with an SSRI who have been prescribed an SSRI* |
|  | *Percentage of patients with a panic disorder with an indication for treatment with a TCA, who have been prescribed a TCA* |
|  | *Percentage of patients with a panic disorder with (moderately) severe agoraphobia with an indication for treatment with in vivo exposure, who have been offered in vivo exposure* |
|  | *Percentage of patients with a panic disorder with (moderately) severe agoraphobia who have been offered in vivo exposure in combination with a pharmacotherapeutic treatment* |
|  | *Percentage of patients with a panic disorder without (or with only mild) agoraphobia with an indication for treatment with panic-management training, who have been offered panic-management training* |
| *Social phobia* | *Percentage of patients with a social phobia of the generalized subtype with an indication for treatment with an SSRI, who have been prescribed an SSRI* |
|  | *Percentage of patients with a social phobia of the generalized subtype with an indication for monotherapy with a benzodiazepine, who have been prescribed monotherapy with a benzodiazepine* |
|  | *Percentage of patients with a social phobia of the specific subtype with an indication for treatment with a beta blocker, who have been prescribed a beta blocker* |
|  | *Percentage of patients with a social phobia with an indication for treatment with in vivo exposure, who have been offered in vivo exposure* |
|  | *Percentage of patients with a social phobia with an indication for cognitive therapy, who have been offered cognitive therapy* |
|  | *Percentage of patients with a social phobia of the generalized subtype with an indication for following a social skills training, who have been offered a social skills training* |
| *Obsessive Compulsive Disorder* | *Percentage of patients with OCD with co morbid severe depression and an indication for treatment with an antidepressant, who have been prescribed one of the antidepressants recommended in the guideline first of all* |
|  | *Percentage of patients with OCD with an indication for treatment with an SSRI, whom have been prescribed an SSRI* |
|  | *Percentage of patients with OCD who did not respond to treatment with a second SSRI with an indication for treatment with an antipsychotic, whose treatment with an SSRI has been supplemented with an antipsychotic* |
|  | *Percentage of patients with OCD with an indication for treatment with clomipramine, who have been prescribed clomipramine* |
|  | *Percentage of patients with OCD with an indication for treatment with exposure and response prevention, who have been offered treatment with exposure and response prevention* |
|  | *Percentage of patients with OCD with an indication for treatment with cognitive therapy, who have been offered cognitive therapy* |
| *Generalized anxiety disorder* | *Percentage of patients with a generalized anxiety disorder with an indication for treatment with paroxetine, who have been prescribed paroxetine* |
|  | *Percentage of patients with a generalized anxiety disorder with an indication for treatment with venlafaxine, who have been prescribed venlafaxine* |
|  | *Percentage of patients with a generalized anxiety disorder with an indication for treatment with buspiron, who have been prescribed buspiron* |
|  | *Percentage of patients with a generalized anxiety disorder with an indication for treatment with cognitive therapy, who have been offered cognitive therapy* |
|  | *Percentage of patients with a generalized anxiety disorder with an indication for treatment with exposure, who have been offered treatment with exposure* |
|  | *Percentage of patients with a generalized anxiety disorder with an indication for treatment with applied relaxation, who have been offered applied relaxation* |
| *PTSD* | *Percentage of patients with PTSD with an indication for treatment with an SSRI, who have been offered an SSRI* |
|  | *Percentage of patients with PTSD with an indication for treatment with a TCA, who have been offered a TCA* |
|  | *Percentage of patients with PTSD with an indication for treatment with EMDR, who have been prescribed EMDR* |
|  | *Percentage of patients with PTSD with an indication for treatment with imaginary exposure, who have been offered treatment with imaginary exposure* |
|  | *Percentage of patients with PTSD with an indication for treatment with cognitive therapy, who have been offered cognitive therapy* |
| *Specific phobia* | *Percentage of patients with a specific phobia with an indication for treatment with exposure, who have been offered treatment with exposure* |
|  | *Percentage of patients with a specific phobia with an indication for treatment with cognitive therapy, who have been offered cognitive therapy* |
| *Hypochondria* | *Percentage of patients with hypochondria with an indication for treatment with cognitive therapy, who have been offered cognitive therapy* |
|  | *Percentage of patients with hypochondria with an indication for treatment with exposure and response prevention, who have been offered treatment with exposure and response prevention* |

**Table B. treatment indicators applicable when pharmacotherapeutic treatment is offered**

| *Percentage of patients with an anxiety disorder who have been prescribed a certain type of medication (e.g. a TCA), where it has been decided to prescribe one of the medications recommended in the guideline (e.g. Clomipramine).* |
| --- |
| *Percentage of patients with an anxiety disorder who have been treated with one of the recommended medications and who have been prescribed the medication according to the target dosage* |
| *Percentage of patients with an anxiety disorder who have been treated with one of the recommended medications and who had been prescribed the medication for the recommended number of weeks before the effects of the treatment were evaluated* |

**Table C. treatment indicators applicable when a form of exposure is offered**

| *Percentage of patients with an anxiety disorder who have been offered a form of exposure, and who were given an explanation of the treatment before it began* |
| --- |
| *Percentage of patients with an anxiety disorder to whom a form of exposure has been offered and who have been given exposure homework assignments on a consistent basis* |
| *Percentage of patients with an anxiety disorder who have been offered a form of exposure and in whom the effect of the treatment was evaluated after they had received it for the recommended number of weeks* |

**Table D. treatment indicators which are applicable when cognitive therapy is offered**

| *Percentage of patients with an anxiety disorder who have been offered a form of cognitive therapy, and who were given an explanation of the treatment before it began* |
| --- |
| *Percentage of patients with an anxiety disorder who have been offered a form of cognitive therapy and who have been given homework assignments associated with this therapy on a consistent basis* |
| *Percentage of patients with an anxiety disorder who have been offered a form of cognitive therapy, and in whom the effect of the treatment was evaluated after they had received it for the recommended number of weeks* |
