# Supplementary material for: Implementing practice guidelines for anxiety disorders in secondary mental health care: a case study
Source: Int J Ment Health Syst. 2012 Sep 20;6:20. doi: 10.1186/1752-4458-6-20 (PMC3499400; doi:10.1186/1752-4458-6-20)
Supplement: Additional file 2 — Questionnaire relating to factors that impede or promote the application of the anxiety disorder guidelines. [file 1752-4458-6-20-S2.doc]

# Additional file 2

# Questionnaire relating to factors that impede or promote the application of the anxiety disorder guidelines

**Instructions:**

The following questionnaire is designed to look at your opinion about using the guideline. This is to determine whether in your view there are any particular problem areas, or whether you see using the guideline as an opportunity. This information will help to determine whether it is worthwhile considering introducing the guideline more widely and, if so, which particular points will require attention. Please tick the answer that corresponds most closely to your own situation and/or opinion.

| 1. | Do you have a copy of the guidelines? |  *yes* |  *no* |  |  |  |
| --- | --- | --- | --- | --- | --- | --- |
| 2. | Do you have a summary of the guidelines? |  *yes* |  *no* |  |  |  |
|  |  | *not at all* | *hardly* | *in part* | *most of it* | *all of it* |
| 3. | To what extent have you read the guidelines? |  |  |  |  |  |
|  |  | *poor* | *limited* | *reasonable* | *good* | *very good* |
| 4. | How do you evaluate your own knowledge of the contents of the guidelines? |  |  |  |  |  |
|  |  | *never* | *sometimes* | *regularly* | *often* | *always* |
| 5. | How often do you currently use the guideline when treating patients? |  |  |  |  |  |
|  | *I think that working according to the guidelines is:* | *Strongly disagree* | *Disagree* | *Neither agree nor disagree* | *Agree* | *Strongly agree* |
| 6. | sensible |  |  |  |  |  |
| 7. | a good thing |  |  |  |  |  |
|  | *To what extent do you agree with the following statements?* | *Strongly disagree* | *Disagree* | *Neither agree nor disagree* | *Agree* | *Strongly agree* |
| 8. | I think that following the guidelines would improve the quality of my work |  |  |  |  |  |
| 9. | My patients think that they should be treated according to the guidelines |  |  |  |  |  |
| 10. | I intend to (continue to) use the guidelines to treat the majority of my patients with an anxiety disorder |  |  |  |  |  |
| 11. | I am able to organize my work in such a way that I can apply these guidelines |  |  |  |  |  |
| 12. | I am able to maintain the chosen treatment approach over several successive treatment meetings with the patient |  |  |  |  |  |
| 13. | Others in my profession think that I should work according to the guideline |  |  |  |  |  |
| 14. | My aim is to try to (continue to) comply with the guidelines for the majority of the patients who I treat |  |  |  |  |  |
|  | *To what extent do you agree with the following statements?* | *Strongly disagree* | *Disagree* | *Neither agree nor disagree* | *Agree* | *Strongly agree* |
| 15. | I am able to apply the guidelines |  |  |  |  |  |
| 16. | Colleagues within my team think that I should work according to the guidelines |  |  |  |  |  |
| 17. | My superiors think that I should work according to the guidelines |  |  |  |  |  |
| 18. | I expect that I will (continue to) follow the recommendations in the guidelines for the majority of patients |  |  |  |  |  |
| 19. | It is difficult for me to adapt my normal working methods in order to use the guidelines |  |  |  |  |  |
| 20. | I think that in most cases, I will (continue to) use the guidelines |  |  |  |  |  |
| 21. | Following the guidelines makes the work of medical service providers more transparent, both for each other and for the outside world |  |  |  |  |  |
| 22. | Within the professional association which I have the most contact with, people think that patients should be treated according to the guidelines |  |  |  |  |  |
| 23. | Patients do not want to be treated according to the approach recommended in the guidelines |  |  |  |  |  |
| 24. | I will (continue to) design most of my treatment according to the recommendations in the guidelines |  |  |  |  |  |
| 25. | I expect that healthcare for patients with an anxiety disorder will improve through the use of the guidelines |  |  |  |  |  |

Original distribution of items:

- 5 items which indicate the extent to which professionals are familiar with the guidelines (1,2,3,4,5)
- 5 items which reflect the ‘attitude’ of professionals to working according to the guidelines (items 6,7,8, 21, 25)
- 5 items which give an impression of the ‘social pressure’ to use the guidelines experienced by professionals (items 9,13,16,17,22)
- 5 items which reflect a professional’s assessment of their ability to adhere to the guidelines in terms of carrying out checks on his/her own actions (items 11, 12,13,15, 19, 23)
- 5 items which give an impression of a professional’s actual intention to (continue to) use the guidelines (items 10,14,18,20,24)
